# Writing a discussion section: how to integrate substantive and statistical expertise

**DOI:** 10.1186/s12874-018-0490-1

**Published:** 2018-04-17

**Authors:** Michael Höfler, John Venz, Sebastian Trautmann, Robert Miller

**Affiliations:** 1 Institute of Clinical Psychology and Psychotherapy, Technische Universität Dresden, Dresden, Germany; 2 Behavioral Epidemiology, Institute of Clinical Psychology and Psychotherapy, Technische Universität Dresden, Dresden, Germany; 3 Faculty of Psychology, Technische Universität Dresden, Dresden, Germany; 4 Department of Medical Epidemiology and Biostatistics, Karolinska Institute, Stockholm, Sweden; 5 Chair of Clinical Psychology and Behavioural Neuroscience, Institute of Clinical Psychology and Psychotherapy, Technische Universität Dresden, Dresden, Germany

**Keywords:** Discussion, Conclusion, Writing, Bias, Causality, Mechanism, Assumptions, Statistician, Substantive researcher, Bayes

## Abstract

**Background:**

When discussing results medical research articles often tear substantive and statistical (methodical) contributions apart, just as if both were independent. Consequently, reasoning on bias tends to be vague, unclear and superficial. This can lead to over-generalized, too narrow and misleading conclusions, especially for causal research questions.

**Main body:**

To get the best possible conclusion, substantive and statistical expertise have to be integrated on the basis of reasonable assumptions. While statistics should raise questions on the mechanisms that have presumably created the data, substantive knowledge should answer them. Building on the related principle of Bayesian thinking, we make seven specific and four general proposals on writing a discussion section.

**Conclusion:**

Misinterpretation could be reduced if authors explicitly discussed what can be concluded under which assumptions. Informed on the resulting *conditional* conclusions other researchers may, according to their knowledge and beliefs, follow a particular conclusion or, based on other conditions, arrive at another one. This could foster both an improved debate and a better understanding of the mechanisms behind the data and should therefore enable researchers to better address bias in future studies.

## Background

After a research article has presented the substantive background, the methods and the results, the discussion section assesses the validity of results and draws conclusions by interpreting them. The discussion puts the results into a broader context and reflects their implications for theoretical (e.g. etiological) and practical (e.g. interventional) purposes. As such, the discussion contains an article’s last words the reader is left with.

Common recommendations for the discussion section include general proposals for writing [1] and structuring (e.g. with a paragraph on a study’s strengths and weaknesses) [2], to avoid common statistical pitfalls (like misinterpreting non-significant findings as true null results) [3] and to “go beyond the data” when interpreting results [4]. Note that the latter includes much more than comparing an article’s results with the literature. If results and literature are consistent, this might be due to shared bias only. If they are not consistent, the question arises why inconsistency occurs – maybe because of bias acting differently across studies [5–7]. Recommendations like the CONSORT checklist do well in demanding all quantitative information on design, participation, compliance etc. to be reported in the methods and results section and “addressing sources of potential bias”, “limitations” and “considering other relevant evidence” in the discussion [8, 9]. Similarly, the STROBE checklist for epidemiological research demands “a cautious overall interpretation of results” and "discussing the generalizability (external validity)" [10, 11]. However, these guidelines do not clarify *how* to deal with the complex bias issue, and how to get to and report conclusions.

Consequently, suggestions on writing a discussion often remain vague by hardly addressing the role of the assumptions that have (often implicitly) been made when designing a study, analyzing the data and interpreting the results. Such assumptions involve mechanisms that have created the data and are related to sampling, measurement and treatment assignment (in observational studies common causes of factor and outcome) and, as a consequence, the bias this may produce [5, 6]. They determine whether a result allows only an associational or a causal conclusion. Causal conclusions, if true, are of much higher relevance for etiology, prevention and intervention. However, they require much stronger assumptions. These have to be fully explicit and, therewith, essential part of the debate since they always involve subjectivity. Subjectivity is unavoidable because the mechanisms behind the data can never be fully estimated from the data themselves [12].

In this article, we argue that the conjunction of substantive and statistical (methodical) knowledge in the verbal integration of results and beliefs on mechanisms can be greatly improved in (medical) research papers. We illustrate this through the personal roles that a statistician (i.e. methods expert) and a substantive researcher should take. Doing so, we neither claim that usually just two people write a discussion, nor that one person lacks the knowledge of the other, nor that there were truly no researchers that have both kinds of expertise. As a metaphor, the division of these two roles into two persons describes the necessary integration of knowledge via the mode of a dialogue. Verbally, it addresses the finding of increased specialization of different study contributors in biomedical research. This has teared apart the two processes of statistical compilation of results and their verbal integration [13]. When this happens a statistician alone is limited to a study’s conditions (sampled population, experimental settings etc.), because he or she is unaware of the conditions’ generalizability. On the other hand, a A substantive expert alone is prone to over-generalize because he or she is not aware of the (mathematical) prerequisites for an interpretation.

The article addresses both (medical) researchers educated in basic statistics and research methods and statisticians who cooperate with them. Throughout the paper we exemplify our arguments with the finding of an association in a cross-tabulation between a binary X (factor) and a binary Y (outcome): those who are exposed to or treated with X have a statistically significantly elevated risk for Y as compared to the non-exposed or not (or otherwise) treated (for instance via the chi-squared independence test or logistic regression). Findings like this are frequent and raise the question which more profound conclusion is valid under what assumptions. Until some decades ago, statistics has largely avoided the related topic of causality and instead limited itself on describing observed distributions (here a two-by-two table between D = depression and LC = lung cancer) with well-fitting models.

**Fig. 1:**
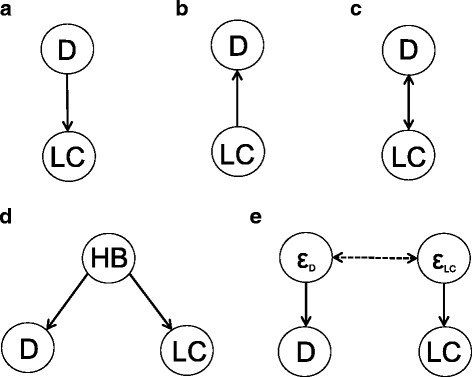
Different conclusions about an association between D and LC. **a** D causes LC, **b** LC causes B, **c** D and LC cause each other, **d** D and LC are associated because of a shared factor (HB), **e** D and LC are associated because they have correlated errors

We illustrate our arguments with the concrete example of the association found between the factor depression (D) and the outcome lung cancer (LC) [14]. Yet very different mechanisms could have produced such an association [7], and assumptions on these lead to the following fundamentally different conclusions (Fig. 1):

- D causes LC (e.g. because smoking might constitute “self-medication” of depression symptoms)
- LC causes D (e.g. because LC patients are demoralized by their diagnosis)
- D and LC cause each other (e.g. because the arguments in both a. and b. apply)
- D and LC are the causal consequence of the same factor(s) (e.g. poor health behaviors - HB)
- D and LC only share measurement error (e.g. because a fraction of individuals that has either depression or lung cancer denies both in self-report measures).

Note that we use the example purely for illustrative purposes. We do not make substantive claims on what of a. through e. is true but show how one should *reflect* on mechanisms in order to find the right answer. Besides, we do not consider research on the D-LC relation apart from the finding of association [14].

Assessing which of a. through e. truly applies requires substantive assumptions on mechanisms: the temporal order of D and LC (a causal effect requires that the cause occurs *before* the effect), shared factors, selection processes and measurement error. Questions on related mechanisms have to be brought up by statistical consideration, while substantive reasoning has to address them. Together this yields provisional assumptions for inferring that are subject to readers’ substantive consideration and refinement. In general, the integration of prior beliefs (anything beyond the data a conclusion depends on) and the results from the data themselves is formalized by Bayesian statistics [15, 16]. This is beyond the scope of this article, still we argue that Bayesian *thinking* should govern the process of drawing conclusions.

Building on this idea, we provide seven specific and four general recommendations for the cooperative process of writing a discussion. The recommendations are intended to be suggestions rather than rules. They should be subject to further refinement and adjustment to specific requirements in different fields of medical and other research. Note that the order of the points is not meant to structure a discussion’s writing (besides 1.).

## Recommendations for writing a discussion section

### Specific recommendations

**Fig. 2:**
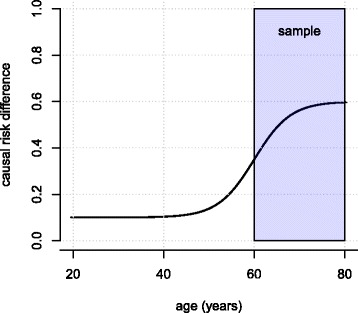
If higher age is related to a larger effect (risk difference) of D on LC, a larger effect estimate is expected in an elder sample
  However, the statistician might add, if effect modification is weak, or the difference in the age distributions is modest (e.g. mean 54 vs. 52 years), selection is unlikely to have produced large (here: upward) bias. In turn, another substantive researcher, who reads the resulting discussion, might instead assume a *decrease* of effect with increasing age and thus infer *downward* bias.
  In practice, researchers should be extremely sensitive for bias due to selection if a sample has been drawn conditionally on a *common consequence* of factor and outcome or a variable associated with such a consequence [19 and references therein]. For instance, hospitalization might be influenced by both D and LC, and thus sampling from hospitals might introduce a false association or change an association’s sign; particularly D and LC may appear to be negatively associated although the association is positive in the general population (Fig. 3).

1. *Start the discussion with the conclusion your design and results unambiguously allow*
  Consider the example on the association between D and LC. Rather than starting with an in-depth (causal) interpretation a finding should firstly be taken as what it allows inferring without doubt: Under the usual assumptions that a statistical model makes (e.g. random sampling, independence or certain correlation structure between observations [17]), the association indicates that D (strictly speaking: measuring D) predicts an elevated LC risk (strictly speaking: measuring LC) in the population that one has managed to sample (source population). Assume that the sample has been randomly drawn from primary care settings. In this case the association is useful to recommend medical doctors to better look at an individual’s LC risk in case of D. If the association has been adjusted for age and gender (conveniently through a regression model), the conclusion modifies to: If the doctor knows a patient’s age and gender (what should always be the case) D has additional value in predicting an elevated LC risk.
2. *Mention the conclusion(s) that researchers would like to draw*
  In the above example, a substantive researcher might want to conclude that D and LC are associated *in a general population* instead of just inferring to patients in primary care settings (a.). Another researcher might even take the finding as evidence for D being a *causal* factor in the etiology of LC, meaning that prevention of D could reduce the incidence rate of LC (in whatever target population) (b.). In both cases, the substantive researcher should insist on assessing the desired interpretation that goes beyond the data [4], but the statistician immediately needs to bring up the next point.
3. *Specify all assumptions to interprete the observed result in the desired (causal) way*
  The explanation of all the assumptions that lead from a data result to a conclusion enables a reader to assess whether he or she agrees with the authors’ inference or not. These conditions, however, often remain incomplete or unclear, in which case the reader can hardly assess whether he or she follows a path of argumentation and, thus, shares the conclusion this path leads to.
  Consider conclusion a. and suppose that, instead of representative sampling in a general population (e.g. all U.S. citizens aged 18 or above), the investigators were only able to sample in primary care settings. Extrapolating the results to another population than the source population requires what is called “external validity”, “transportability” or the absence of “selection bias” [18, 19]. No such bias occurs if the parameter of interest is equal in the source and the target population. Note that this is a weaker condition than the common belief that the sample must represent the target population in *everything*. If the parameter of interest is the difference in risk for LC between cases and non-cases of D, the condition translates into: the risk difference must be equal in target and source population.
  For the causal conclusion b., however, sufficient assumptions are very strict. In an RCT, the conclusion is valid under random sampling from the target population, random allocation of X, perfect compliance in X, complete participation and no measurement error in outcome (for details see [20]). In practice, on the other hand, the derivations from such conditions might sometimes be modest what may produce little bias only. For instance, non-compliance in a specific drug intake (treatment) might occur only in a few individuals to little extent through a random process (e.g. sickness of a nurse being responsible for drug dispense) and yield just small (downward) bias [5]. The conclusion of *downward* bias might also be justified if non-compliance does not cause anything that has a larger effect on a Y than the drug itself. Another researcher, however, could believe that non-compliance leads to taking a more effective, alternative treatment. He or she could infer *upward* bias instead if well-informed on the line of argument.
4. *Otherwise avoid causal language*
  In practice, researchers frequently use causal language yet without mentioning any assumptions. This does not imply that they truly have a causal effect in mind, often causal and associational wordings are carelessly used in synonymous way. For example, concluding “depression increases the risk of lung cancer” constitutes already causal wording because it implies that a change in the depression status would change the cancer risk. Associational language like “lung cancer risk is elevated if depression occurs”, however, would allow for an elevated lung cancer risk in depression cases just because LC and D share some causes (“inducing” or “removing” depression would not change the cancer risk here).
5. *Reflect critically on how deviations from the assumptions would have influenced the results*
  Often, it is unclear where the path of argumentation from assumptions to a conclusion leads when alternative assumptions are made. Consider again bias due to selection. A different effect in target and source population occurs if effect-modifying variables distribute differently in both populations. Accordingly, the statistician should ask which variables influence the effect of interest, and whether these can be assumed to distribute equally in the source population and the target population. The substantive researcher might answer that the causal risk difference between D and LC likely increases with age. Given that this is true, and if elder individuals have been oversampled (e.g. because elderly are over-represented in primary care settings), both together would conclude that sampling has led to over-estimation (despite other factors, Fig. 2).

**Fig. 3:**
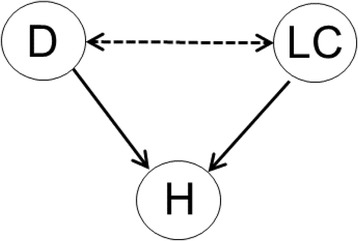
If hospitalization (H) is a common cause of D and LC, sampling conditionally on H can introduce a spurious association between D and LC ("conditioning on a collider")
6. *Comment on all main types of bias and the inferential consequences they putatively have*
  Usually, only some kinds of bias are discussed, while the consequences of others are ignored [5]. Besides selection the main sources of bias are often measurement and confounding. If one is only interested in association, confounding is irrelevant. For causal conclusions, however, assumptions on all three kinds of bias are necessary.
  Measurement error means that the measurement of a factor and/or outcome deviates from the true value, at least in some individuals. Bias due to measurement is known under many other terms that describe the reasons why such error occurs (e.g. “recall bias” and “reporting bias”). In contrast to conventional wisdom, measurement error does not always bias association and effect estimates downwards [5, 6]. It does, for instance, if only the factor (e.g. depression) is measured with error and the errors occur independently from the outcome (e.g. lung cancer), or vice versa (“non-differential misclassification”) [22 and references therein]. However, many lung cancer cases might falsely report depression symptoms (e.g. to express need for care). Such false positives (non-cases of depression classified as cases) may also occur in non-cases of lung cancer but to a lesser extent (a special case of “differential misclassification”). Here, bias might be upward as well. Importantly, false positives cause larger bias than false negatives (non-cases of depression falsely classified as depression cases) as long as the relative frequency of a factor is lower than 50% [21]. Therefore, they should receive more attention in discussion. If measurement error occurs in depression *and* lung cancer, the direction of bias also depends on the correlation between both errors [21].
  Note that what is in line with common standards of “good” measurement (e.g. a Kappa value measuring validity or reliability of 0.7) might anyway produce large bias. This applies to estimates of prevalence, association and effect. The reason is that while indices of measurement are one-dimensional, bias depends on two parameters (sensitivity and specificity) [21, 22]. Moreover, estimates of such indices are often extrapolated to different kinds of populations (typically from a clinical to general population), what may be inadequate. Note that the different kinds of bias often interact, e.g. bias due to measurement might depend on selection (e.g. measurement error might differ between a clinical and a general population) [5, 6].
  Assessment of bias due to confounding variables (roughly speaking: common causes of factor and outcome) requires assumptions on the entire system of variables that affect both factor and outcome. For example, D and LC might share several causes such as stressful life events or socioeconomic status. If these influence D and LC with the same effect direction, this leads to overestimation, otherwise (different effect directions) the causal effect is underestimated. In the medical field, many unfavorable conditions may be positively related. If this holds true for all common factors of D and LC, upward bias can be assumed. However, not all confounders have to be taken into account. Within the framework of “causal graphs”, the “backdoor criterion” [7] provides a graphical rule for sets of confounders to be sufficient when adjusted for. Practically, such a causal graph must include all factors that directly or indirectly affect both D and LC. Then, adjustment for a set of confounders that meets the “backdoor criterion” in the graph completely removes bias due to confounding. In the example of Fig. 4 it is sufficient to adjust for Z_1_ and Z_2_ because this “blocks” all paths that otherwise lead backwards from D to LC. Note that fully eliminating bias due to confounding also requires that the confounders have been collected without measurement error [5, 6, 23]. Therefore, the advice is always to concede at least some “residual” bias and reflect on the direction this might have (could be downward if such error is not stronger related to D and LC than a confounder itself).
  Whereas the statistician should pinpoint to the mathematical insight of the backdoor criterion, its application requires profound substantive input and literature review. Of course, there are numerous relevant factors in the medical field. Hence, one should practically focus on those with the highest prevalence (a very seldom factor can hardly cause large bias) and large assumed effects on both X and Y.

**Fig. 4:**
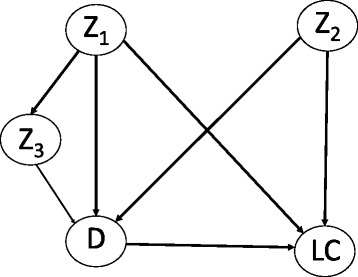
Causal graph for the effect of D on LC and confounders Z_1_, Z_2_ and Z_3_
  If knowledge on any of the three kinds of bias is poor or very uncertain, researchers should admit that this adds uncertainty in a conclusion: systematic error on top of random error. In the Bayesian framework, quantitative bias analysis formalizes this through the result of larger variance in an estimate. Technically, this additional variance is introduced via the variances of distributions assigned to “bias parameters”; for instance a misclassification probability (e.g. classifying a true depression case as non-case) or the prevalence of a binary confounder and its effects on X and Y. Of course, bias analysis also changes point estimates (hopefully reducing bias considerably). Note that conventional frequentist analysis, as regarded from the Bayesian perspective, assumes that all bias parameters were zero with a probability of one [5, 6, 23]. The only exceptions (bias addressed in conventional analyses) are adjustment on variables to hopefully reduce bias due to confounding and weighting the individuals (according to variables related to participation) to take into account bias due to selection.
  If the substantive investigator understands the processes of selection, measurement and confounding only poorly, such strict analysis numerically reveals that little to nothing is known on the effect of X on Y, no matter how large an observed association and a sample (providing small random error) may be [5, 6, 23]). This insight has to be brought up by the statistician. Although such an analysis is complicated, itself very sensitive to how it is conducted [5, 6] and rarely done, the Bayesian thinking behind it forces researchers to better understand the processes behind the data. Otherwise, he or she cannot make any assumptions and, in turn, no conclusion on causality.
7. *Propose a specific study design that requires less and weaker assumptions for a conclusion*
  Usually articles end with statements that only go little further than the always true but never informative statement “more research is needed”. Moreover, larger samples and better measurements are frequently proposed. If an association has been found, a RCT or other interventional study is usually proposed to investigate causality. In our example, this recommendation disregards that: (1) onset of D might have a different effect on LC risk than an intervention against D (the effect of onset cannot be investigated in any interventional study), (2) the effects of onset and intervention concern different populations (those without vs. those with depression), (3) an intervention effect depends on the mode of intervention [24], and (4) (applying the backdoor criterion) a well-designed observational study may approximatively yield the same result as a randomized study would [25–27]. If the effect of “removing” depression is actually of interest, one could propose an RCT that investigates the effect of treating depression in a strictly defined way and in a strictly defined population (desirably in all who meet the criteria of depression). Ideally, this population is sampled randomly, and non-participants and dropouts are investigated with respect to assumed effect-modifiers (differences in their distributions between participants and non-participants can then be addressed e.g. by weighting [27]). In a non-randomized study, one should collect variables supposed to meet the backdoor-criterion with the best instruments possible.

### General recommendations

Yet when considering 1) through 7); i.e. carefully reflecting on the mechanisms that have created the data, discussions on statistical results can be very misleading, because the basic statistical methods are mis-interpreted or inadequately worded.

(8) *Don’t mistake the absence of evidence as evidence for absence*
  A common pitfall is to consider the lack of evidence for the alternative hypothesis (e.g. association between D and LC) as evidence for the null hypothesis (no association). In fact, such inference requires an a-priori calculated sample-size to ensure that the type-two error probability does not exceed a pre-specified limit (typically 20% or 10%, given the other necessary assumptions, e.g. on the true magnitude of association). Otherwise, the type-two error is unknown and in practice often large. This may put a “false negative result” into the scientific public that turns out to be “unreplicable” – what would be falsely interpreted as part of the “replication crisis”. Such results are neither positive nor negative but *uninformative*. In this case, the wording “there is no evidence for an association” is adequate because it does not claim that there is no association.
(9) *Strictly distinguish between discussing pre-specified hypotheses and newly proposed hypotheses from post-hoc analyses*
  Frequently, it remains unclear which hypotheses have been a-priori specified and which have been brought up only after some data analysis. This, of course, is scientific malpractice because it does not enable the readership to assess the random error emerging from explorative data analysis. Accordingly, the variance of results across statistical methods is often misused to filter out the analysis that yields a significant result (“*p*-hacking”, [28]). Pre-planned tests (via writing a grant) leave at least less room for p-hacking because they specify a-priori which analysis is to be conducted.
  On the other hand, post-hoc analyses can be extremely useful for identifying unexpected phenomena and creating new hypotheses. Verbalization in the discussion section should therefore sharply separate between conclusions from hypothesis testing and *new hypotheses* created from data exploration. The distinction is profound, since a newly proposed hypothesis just makes a new claim. Suggesting new hypotheses cannot be wrong, this can only be inefficient if many hypotheses turn out to be wrong. Therefore, we suggest proposing only a limited number of new hypotheses that appear promising to stimulate further research and scientific progress. They are to be confirmed or falsified with future studies. A present discussion, however, should yet explicate the testable predictions a new hypothesis entails, and how a future study should be designed to keep bias in related analyses as small as possible.
  Confidence intervals address the problem of reducing results to the dichotomy of significant and non-significant through providing a range of values that are compatible with the data at the given confidence level, usually 95% [29].
  This is also addressed by Bayesian statistics that allows calculating what frequentist *p*-values are often misinterpreted to be: the probability that the alternative (or null) hypothesis is true [17]. Moreover, one can calculate how likely it is that the parameter lies within any specified range (e.g. the risk difference being greater than .05, a lower boundary for practical significance) [15, 16]. To gain these benefits, one needs to specify how the parameter of interest (e.g. causal risk difference between D and LC) is distributed *before* inspecting the data. In Bayesian statistics (unlike frequentist statistics) a parameter is a random number that expresses prior beliefs via a “prior distribution”. Such a “prior” is combined with the data result to a “posterior distribution”. This integrates both sources of information.
  Note that confidence intervals also can be interpreted from the Bayesian perspective (then called “credibility interval”). This assumes that all parameter values were equally likely (uniformly distributed, strictly speaking) before analyzing the data [5, 6, 20].
(10) *Do not over-interpret small findings. Statistical significance should not be mis-interpreted as practical significance*
  Testing just for a non-zero association can only yield evidence for an association deviating from zero. A better indicator for the true impact of an effect/association for clinical, economic, political, or research purposes is its magnitude. If an association between D and LC after adjusting for age and gender has been discovered, then the knowledge of D has additional value in predicting an elevated LC probability beyond age and gender. However, there may be many other factors that stronger predict LC and thus should receive higher priority in a doctor’s assessment. Besides, if an association is small, it may yet be explained by modest (upward) bias. Especially large samples often yield significant results with little practical value. The *p*-value does not measure strength of association [17]. For instance, in a large sample, a Pearson correlation between two dimensional variables could equal 0.1 only but with a *p*-value <.001. A further problem arises if the significance threshold of .05 is weakened post-hoc to allow for “statistical trends” (*p* between .05 and .10) because a result has “failed to reach significance” (this wording claims that there is truly an association. If this was known, no research would be necessary).
  It is usually the statistician’s job to insist not only on removing the attention from pure statistical significance to confidence intervals or even Bayesian interpretation, but also to point out the necessity of a meaningful cutoff for practical significance. The substantive researcher then has to provide this cutoff.
(11) *Avoid claims that are not statistically well-founded*
  Researchers should not draw conclusions that have not been explicitly tested for. For example, one may have found a positive association between D and LC (e.g. *p* = .049), but this association is not significant (e.g. *p* = .051), when adjusting for “health behavior”. This does not imply that “health behavior” “explains” the association (yet fully). The difference in magnitude of association in both analyses compared here (without and with adjustment on HB) may be very small and the difference in *p*-values (“borderline significance” after adjustment) likely to emerge from random error. This often applies to larger differences in *p* as well.
  Investigators, however, might find patterns in their results that they consider worth mentioning for creating hypotheses. In the example above, adding the words “in the sample”, would clarify that they refer just to the difference of two *point estimates*. By default, “association” in hypotheses testing should mean “statistically significant association” (explorative analyses should instead refer to “suggestive associations”).

## Conclusions

Some issues of discussing results not mentioned yet appear to require only substantive reasoning. For instance, Bradford Hill’s consideration on “plausibility” claims that a causal effect is more likely, if it is in line with biological (substantive) knowledge, or if a dose-response relation has been found [30]. However, the application of these considerations itself depends on the trueness of assumptions. For instance, bias might act differently across the dose of exposure (e.g. larger measurement error in outcome among those with higher dosage). As a consequence, a pattern observed across dose may mask a true or pretend a wrong dose-response relation [30]. This again has to be brought up by statistical expertise.

There are, however, some practical issues that hinder the cooperation we suggest. First, substantive researchers often feel discomfort when urged to make assumptions on the mechanisms behind the data, presumably because they fear to be wrong. Here, the statistician needs to insist: “If you are unable to make any assumptions, you cannot conclude anything!” And: “As a scientist you have to understand the processes that create your data.” See [31] for practical advice on how to arrive at meaningful assumptions.

Second, statisticians have long been skeptical against causal inference. Still, most of them focus solely on describing observed data with distributional models, probably because estimating causal effects has long been regarded as unfeasible with scientific methods. Training in causality remains rather new, since strict mathematical methods have been developed only in the last decades [7].

The cooperation could be improved if education in both fields focused on the insight that one cannot succeed without the other. Academic education should demonstrate that in-depth conclusions from data unavoidably involve prior beliefs. Such education should say: Data do not “speak for themselves”, because they “speak” only ambiguously and little, since they have been filtered through various biases [32]. The subjectivity introduced by addressing bias, however, unsettles many researchers. On the other hand, conventional frequentist statistics just pretends to be objective. Instead of accepting the variety of possible assumptions, it makes the absurd assumption of “no bias with probability of one”. Or it avoids causal conclusions at all if no randomized study is possible. This limits science to investigating just associations for all factors that can never be randomized (e.g. onset of depression). However, the alternative of Bayesian statistics and thinking are themselves prone to fundamental cognitive biases which should as well be subject of interdisciplinary teaching [33].

Readers may take this article as an invitation to read further papers’ discussions differently while evaluating our claims. Rather than sharing a provided conclusion (or not) they could ask themselves whether a discussion enables them to clearly specify *why* they share it (or not). If the result is uncertainty, this might motivate them to write their next discussion differently. The proposals made in this article could help shifting scientific debates to where they belong. Rather than arguing on misunderstandings caused by ambiguity in a conclusion’s assumptions one should argue on the assumptions themselves.

## Abbreviations

D: depression
HB: health behavior
LC: lung cancer
RCT: randomized clinical trial
X: factor variable
Y: outcome variable
